# Ultrafast electron microscopy integrated with a direct electron detection camera

**DOI:** 10.1063/1.4983226

**Published:** 2017-05-08

**Authors:** Young Min Lee, Young Jae Kim, Ye-Jin Kim, Oh-Hoon Kwon

**Affiliations:** 1 Department of Chemistry, School of Natural Science, Ulsan National Institute of Science and Technology (UNIST), 50 UNIST-gil, Ulsan 44919, South Korea; 2 Center for Soft and Living Matter, Institute for Basic Science (IBS), 50 UNIST-gil, Ulsan 44919, South Korea

## Abstract

In the past decade, we have witnessed the rapid growth of the field of ultrafast electron microscopy (UEM), which provides intuitive means to watch atomic and molecular motions of matter. Yet, because of the limited current of the pulsed electron beam resulting from space-charge effects, observations have been mainly made to periodic motions of the crystalline structure of hundreds of nanometers or higher by stroboscopic imaging at high repetition rates. Here, we develop an advanced UEM with robust capabilities for circumventing the present limitations by integrating a direct electron detection camera for the first time which allows for imaging at low repetition rates. This approach is expected to promote UEM to a more powerful platform to visualize molecular and collective motions and dissect fundamental physical, chemical, and materials phenomena in space and time.

Atomic level description of structures is central to the understanding of chemical, physical, materials, and biological phenomena. Now, there exist a range of well-established experimental techniques including X-ray/neutron diffraction, NMR, and electron microscopy for determining the equilibrium structures of molecules, solids, and biological architectures. Many processes, however, often appear complex because we watch them on an extended timescale, during which the observation of serial steps in the processes is integrated in time. For example, the ultimate timescale for chemical bonds to form and break is that of a single vibrational period, tens to hundreds of femtoseconds (fs), at the length scale of a few Å.^1^ On the other hand, in functional assemblies of atoms and molecules, localized processes involving electron-phonon (e-ph) and phonon-phonon interactions occur in up to a few tens of picoseconds (ps).^2^ Ensuing events of acoustic phonon generation, strain propagation, and structural phase/conformation changes can develop at longer times spanning ps to milliseconds (ms) depending on the size of field of view.^3–6^

In this regard, there have been several approaches for transient-structural characterization on the atomic/molecular timescale.^7^ The development of time-resolved diffraction techniques, both the X-ray and electron, with ultrafast temporal resolution has opened up a direct window on the time-evolving nuclear configuration of molecules and solids during photo-induced structural dynamics in atomic spacings (Fourier space) for periodic (crystalline) structures.^8–11^ For fleeting macromolecules (biological or abiological) and single particles, non-periodic (noncrystalline) details need to be captured from real-space imaging. Accordingly, recent efforts have been bifurcated to developing/modifying electron microscopes with laser-driven photocathodes, referred to as ultrafast electron microscopy (UEM), to take pictures of fine spatial resolution and high temporal resolutions (better than the video rate of ms).^12–17^ The applications of the methodology have shown promises in the repertoire of fields; some of the examples are the near-field imaging of photon-electron interactions on nanostructures,^18,19^ electronic dynamics correlated with atomic and molecular structural evolution,^20^ the phonon and lattice dynamics of atomic/molecular assemblies,^2,3^ mechanical motions,^4,21–23^ phase transformation in solids,^5,6,24–26^ and carrier dynamics.^27,28^

In UEM, pulsed lasers of fs or longer temporal durations are used to excite a specimen (clocking dynamics therein) and to illuminate a photocathode for generating a pulsed electron beam as a “pump-probe” scheme in ultrafast spectroscopy.^7,29^ Two different modes of operation are emerging. One is the stroboscopic imaging, in which the optical-pump electron-probe pulses are repetitively cycled for integration to build a time-resolved image, a diffraction pattern, or an electron-energy-loss spectrum at each time delay between the pump and probe. The other is the single-shot imaging, which uses a single optical pulse and multiple sequential probe electron pulses, intense enough for each pulse to produce an image (and a diffraction pattern) from a single photoinduced event. A spatial resolution approaching several Å has been reported with the stroboscopic method.^2,30^

Yet, the stroboscopic mode using fs photoelectron packets suffers from a significant problem, Coulomb repulsion between charged particles confined in space and time. This space-charge effect limits the formation and propagation of fs-long electron pulses considerably; ultrashort electron pulses broaden in space and lengthen in time.^30,31^ Typically, the spatial resolution of pulsed electron packets generated by optical pulses of a few hundreds of femtoseconds at lower repetition rates than MHz seems to reside around several nanometers at best.^32^ To surmount the limitation, the approach has been the optimization of a gun region to generate space-charge minimized photoelectron packets. In this space-charge quasi-free regime, the number of electrons in each fs pulse has to be a few to a few dozens depending on their density. To build up an image of affordably good quality integrated in seconds, a stroboscopic measurement at a repetition rate of ∼1 MHz is typical for detecting at least tens of electrons per pixel on a conventional charge-coupled device (CCD) camera. In previous reports from other groups, the 0.34 nm lattice fringes of graphitized carbon were resolved when pulsed photoelectron packets were generated at the repetition rate of 25 MHz.^2^ Recently, the image of gold crystalline nanoparticles with the lattice fringes of 0.23 nm was successfully taken at 2 MHz.^30^ This high repetition, however, narrows the choice of a specimen because the specimen upon photoexcitation must fully revert to its original configuration in less than 1 microsecond (*μ*s), the case of which is practically rare. Recently, stroboscopic imaging at as low as 25 kHz has been reported.^3^

Toward the wider scope of the stroboscopic imaging, a clocking period has to be set long enough to ensure the full relaxation of a specimen in each cycle of excitation. Moreover, the number of electrons per probe pulse is desired to reside in the space-charge free regime, and the pulse duration needs to be brief enough to take snapshots of structural changes. Here, we report the development of UEM uniquely equipped with a direct electron-detection camera, for the first time to our best knowledge, which poses much higher sensitivity to incident electrons. This allows for a less number of electrons to be needed to record an image, the case of which translates into the lower repetition rate of stroboscopic imaging possibly expanding the selection of research themes.

Fig. 1 displays the pictorial setup of UEM installed at UNIST. The instrumentation couples three independent laser systems to a transmission electron microscope (TEM) modified in several aspects for both the operational modes of stroboscopy and single-pump multiple-probe. A 200-kV TEM with a LaB_6_ thermionic cathode (JEM-2100 LaB_6_, Jeol) was modified to host two ports for optical access, one leading to a photocathode and the other to a specimen, composed of an optical window and a mirror assembly per each port. Additionally, a weak, extra condenser lens (C0 lens, IDES) is integrated between the electron gun part and the conventional condenser lens system to increase the throughput of photoelectron packets passing through the hole of the laser mirror along the optical axis and the condenser lens system while preserving affordably the coherent beam. The LaB_6_ cathode is replaced with a flat tantalum cathode having diameter of 800 *μ*m. The use of the large, flat photocathode allows for varying the area of its exposure to driving UV laser pulses and, therefore, controlling beam current, which compromises beam coherence and the temporal broadening of the pulsed electron packets to a ps regime.

**FIG. 1.**
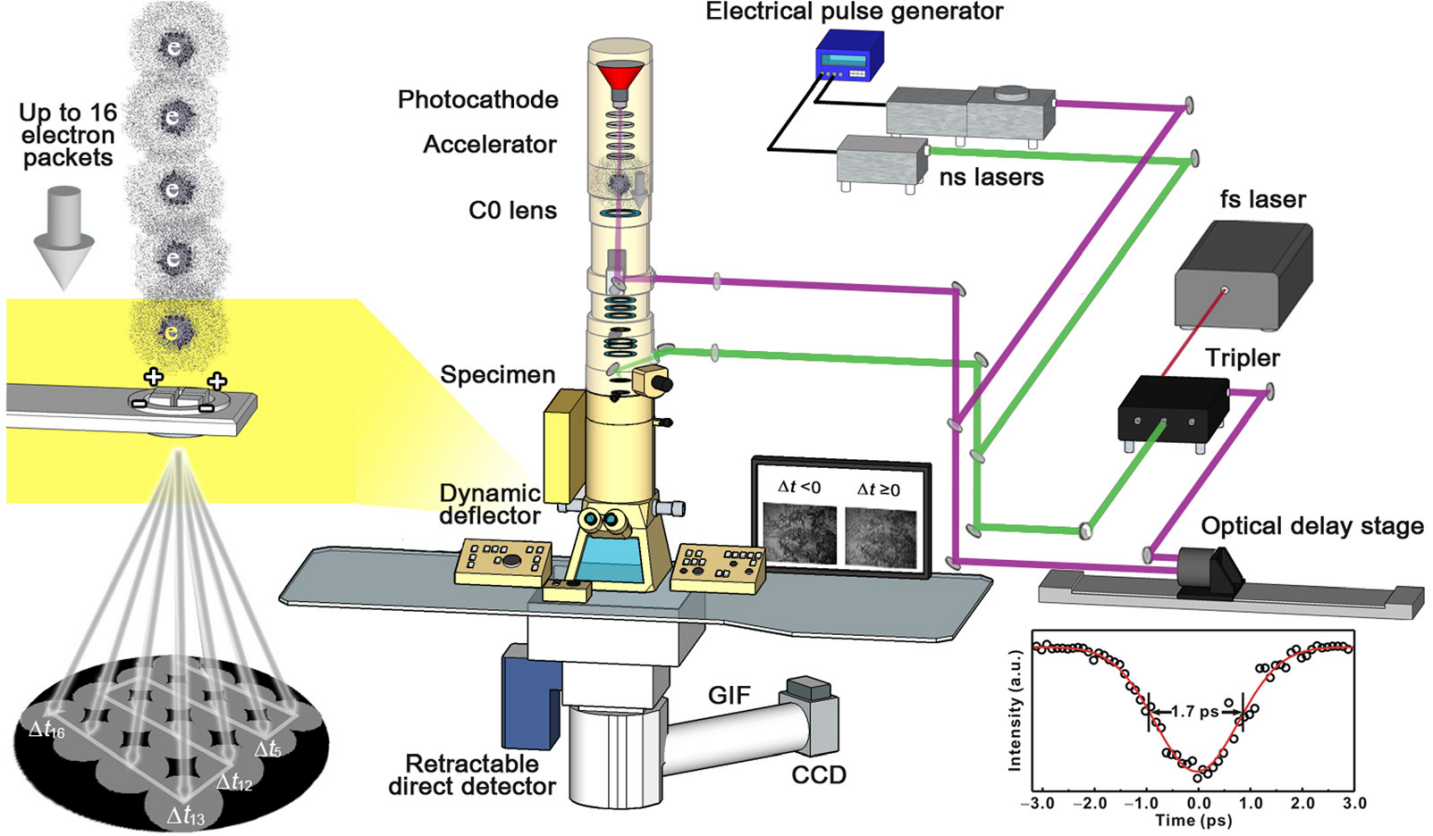
Layout of the UEM setup at UNIST. Three independent laser systems are interfaced to a modified TEM with two optical access ports to direct laser pulses to a photocathode and a specimen, an extra condenser lens introduced between a Wehnelt assembly and a conventional condenser lens section, and the dynamic orthogonal deflector. The unique features are highlighted in magnified panels. The inset shows the profile of the time scan at the zero-loss peak of the EEL spectrum; the FWHM of the temporal duration is indicated inside.

For stroboscopy, an ytterbium-based amplifier (Pharos SP, Light Conversion), which produces ultrashort pulses of up to 30 *μ*J at 1030 nm with a variable repetition rate from a single shot to 600 kHz, and a pair of solid-state diode lasers to cover the dynamic ranges of 100 fs to 4 ns and 1 ns to ms, respectively, are used. The one part of the fs laser output is frequency doubled to 515 nm for initiating the structural dynamics of a specimen. The other part of the output is frequency-quadrupled to 257 nm with the stretchable pulse duration from 170 fs to 10 ps and directed to the photocathode for generating probe electron pulses. The time delay between the optical pump pulse and the probe electron pulse is tunable using an optical delay line via a motorized 60-cm long translational stage. To control the time delay between the two diode lasers of ∼15 ns pulse duration emitting at 266 nm (Awave-266-100mW-5 K, Advanced Optowave) and 532 nm (Awave-532-1 W-5 K, Advanced Optowave) for producing photoelectron packets and exciting a specimen, respectively, an electrical pulse/delay generator (DG535, SRS) is utilized.

The resulting time frames of micrographs and diffractograms were taken by using a retractable direct electron-detection camera (K2 Summit, Gatan), which directly detects incoming electrons without the need of a scintillator. This detection scheme greatly reduces the point spread function and improves the detective quantum efficiency (DQE) at high spatial frequencies and contrast at low frequencies. A high frame rate and a fast detection algorithm (“dose frame” mode) minimize the counting of more than one electron per pixel, eliminating the Landau noise and reducing the coincidence loss. Additionally, the camera enables finding the centroid of the electron peak to subpixel accuracy (“super-resolution” mode). These give high DQE to the direct detection camera, which provides great benefits of taking photoelectron images at low repetition rates. For example, the K2 direct detection camera can have up to 6 times higher DQE than the US4000 CCD camera at a physical Nyquist frequency fraction of 0.2 and even higher DQE at a higher physical Nyquist frequency fraction.^33^ For time-resolved electron-energy-loss spectrum and energy-filtered image measurements, a CCD camera (US4000, Gatan), which is attached to the end of a post-column type imaging filter (GIF Quantum SE, Gatan), was used with the direct detector being retrieved from the optical axis. From the photon-induced near-field electron microscopy (PINEM) imaging of a network of silver nanowires, the instrumental response function was obtained to be 1.7 ps (the inset of Fig. 1). Fig. 2 compares the quality of time-framed images of a test specimen, a gold nanorod (716820, Sigma Aldrich), obtained using the direct detector in the linear integration, counting, and dose-frame modes with that of a time-framed image taken with the CCD camera.

**FIG. 2.**
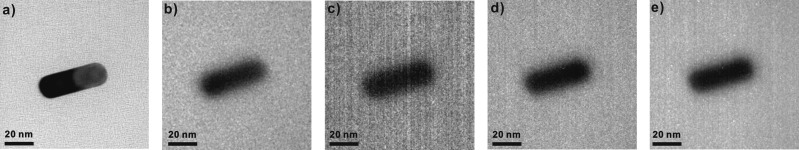
Comparison of bright-field micrographs of the gold nanoparticle acquired in the different modes of the same microscope. (a) Image taken with a continuous-wave thermionic electron beam. (b)–(e) Images using a pulsed electron beam and collected with the US 4000 CCD camera (b) and the K2 Summit direct detector in the linear-integration mode (c), the counting mode (d), and the dose-frame mode (e). The pulsed electron beam was generated by photoelectric effects driven by UV laser pulses of 170-fs temporal duration. Vertical striations are apparent in the images taken with the K2 direct detector, especially in the linear-integration mode (c). This artifact seems to originate from the readout noise as frequently observed under too low or too high dose rates as reported.^39^

For the single-shot mode, an arbitrary waveform generation laser (IDES) replaces the previous ns photocathode-drive laser. The high-power laser consists of a waveform generator that drives a fiber-based electro-optical modulator, which temporally shapes a continuous-wave fiber laser seed pulse, and a series of laser diode heads that amplifies the modulated waveforms having energies of >1 J. The temporal shaping of laser pulses makes it possible for varying electron pulse durations from 10 ns to 250 *μ*s and the production of electron pulse trains with a controllable number of pulses up to 16 and delay among the pulses. Each pulse captures an image of a specimen at a designated time delay. Depending on the arrival time of electron pulses to a dynamic, orthogonal electrostatic deflector assembly located below a set of projection lenses, each pulse containing image or diffraction information experiences different travel trajectory along the GIF column and is registered as a patch on different positions of the CCD camera.^13,24^ Finally, the single entire image is recorded and spatially segmented into a time series of images (Fig. 1).

The demonstrative experiments were performed for a commercial copper grid covered with single-crystalline gold membranes (21420-25, Ted Pella). To initiate structural dynamics, the fluence of the excitation pulse was chosen as 0.4 mJ/cm^2^. The repetition rate of the excitation was set at 10 kHz in a way that the original structural configuration was fully recovered before successive excitation pulses were introduced to the specimen; stroboscopic imaging at 5 kHz was also possible (see supplementary material). In Fig. 3(a), we present time-framed micrographs, each of which was taken using the direct detector in the counting mode and integrated for 5 s, at representative time delays (Δ*t*) with respect to the clocking pulse (Δ*t* = 0). In the first image taken at Δ*t* = −50 ps, bend contours were observed, which appear as dark patches in the image as a result of diffraction contrast effects occurring in warped, thin single crystals of constant thickness. In the dark regions, the zone axis ([100]) is well aligned with the optical axis of the electron beam resulting in efficient diffraction of the incident electron beam, whereas in the lighter regions, the zone axis is off from the optical axis with less scattering efficiency. After Δ*t* **= 0, visual differences emerged in the bend contours. Because bend contours change when tilting of the local crystal lattice occurs by deformation, they offer a sensitive measure of such deformations in images. A series of time-framed micrographs with equal time steps provides a movie of the morphological dynamics. To quantify the time dependence of micrographs, the cross-correlation values between the image at each time delay and a reference image recorded before Δ*t* = 0 were obtained. Long-term specimen drifts deteriorating the cross-correlation values were corrected by the alignment of all time frames with respect to “land mark” features. Over a region of interest, the cross-correlation values were evaluated and found to fit to a single-exponential function with a time constant of 0.3 ns. This indicates that the macroscopic, morphological deformation occurs on the observed timescale.

**FIG. 3.**
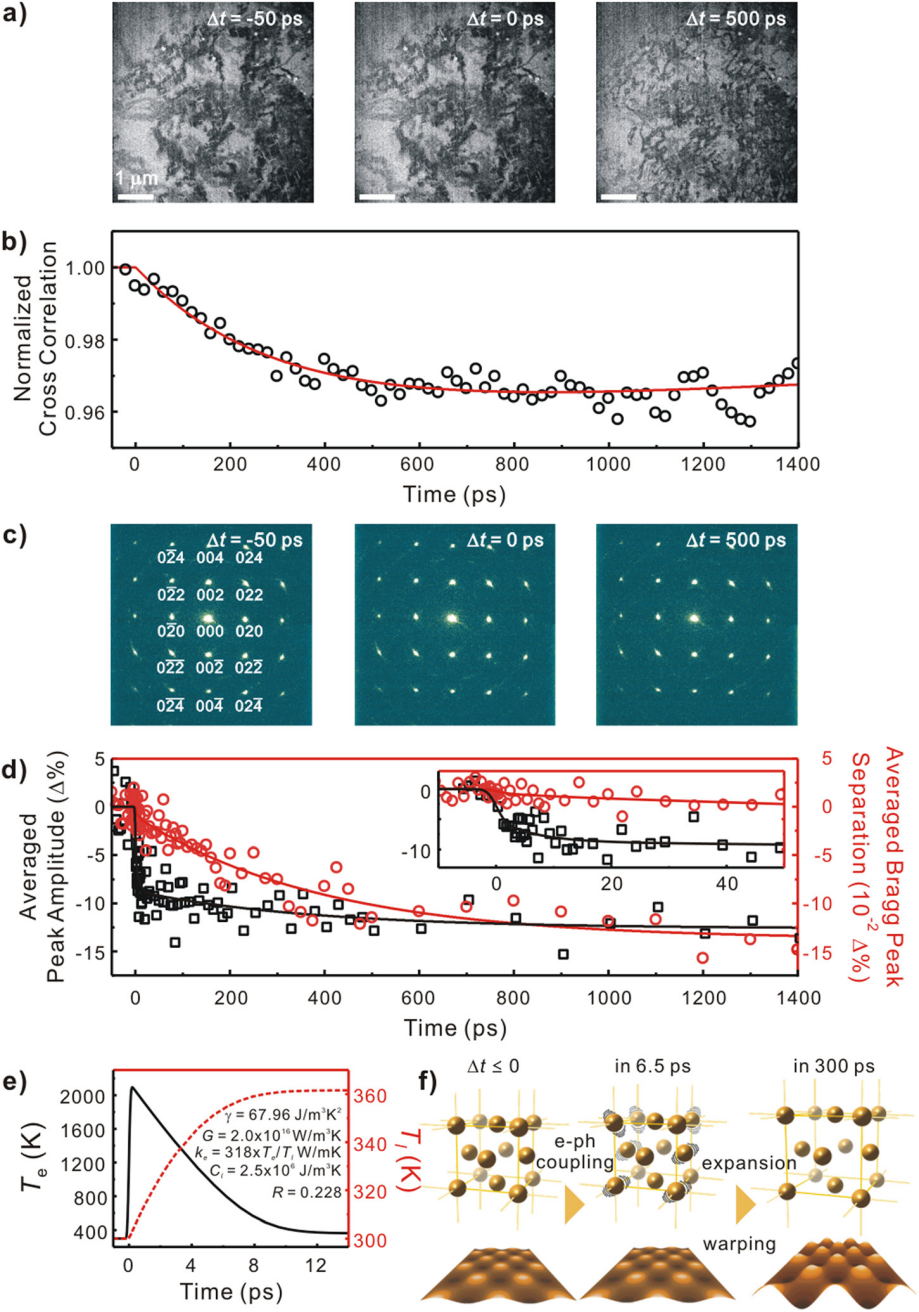
Time-resolved micrographs and diffractograms of a single-crystalline gold membrane. (a) Representative time-framed images obtained with the direct detector in the counting mode. (b) Image cross-correlation profile. (c) Representative time-framed diffraction patterns taken with the conventional CCD. The probed area was 4 *μ*m in diameter. (d) Time dependence of the averaged separation of conjugated Bragg peaks (circle) and the averaged amplitude (square) of selected peaks (indexed inside). (e) Electron temperature (*T_e_*) relaxation at surfaces of a 20 nm thick gold membrane (solid) and lattice (*T_l_*) heating (dotted) as profiled by the TTM. The material constants used were denoted inside the panel. (f) Scheme of atomic-scale motion and morphological change in the single-crystalline gold membrane upon pulsed photoexcitation.

As a complementary set of experiments to this morphological change, time-resolved diffraction measurements further support the observation at the macroscopic scale by revealing the structural dynamics in the lattice. With probing the concentric region up to 4 *μ*m from the center in Fig. 3(a), because the surface normal of the gold membrane is parallel to the zone axis, the diffractograms were indexed by the face-centered-cubic structure as indicated in Fig. 3(c). From the time-resolved analysis of separations between conjugate Bragg spots and the intensity of each Bragg spot, the lattice structural dynamics was tracked as follows: First, because the intensity change of a Bragg spot is related to the thermal motion of atoms about their equilibrium position in a lattice (described by the Debye-Waller factor), the intensity decrease in 6.5 ± 5.8 ps as observed in Fig. 3(d) indicates the timescale of electron-phonon scattering heating up the lattice upon photoexcitation. The electron and lattice temperature profiles during thermal relaxation after pulsed laser heating in this experiment can be simulated by numerically solving the two-temperature model (TTM) as presented in Fig. 3(e).^34,35^ The profiles are generated by two coupled diffusion equations, *C_e_*(*T_e_*)$\frac{\partial T_{e}}{\partial t}=\frac{\partial }{\partial x}$(*k_e_*$\frac{\partial T_{e}}{\partial x}$) − *G*(*T_e_* − *T_l_*) + *S*(*x*, *t*) and *C_l_*(*T_l_*)$\frac{\partial T_{l}}{\partial t}$ = *G*(*T_e_* − *T_l_*), where the laser heat source term is described as *S*(*x*, *t*) = $\frac{\sqrt{\beta }}{\pi }\frac{1\;-\;R}{t_{p}\delta_{s}}$*Φ*$e^{-\;x/\delta_{s}\;-\;\beta {\left((t\;-\;2t_{p})/t_{p}\right)}^{2}}$; *C_e_* is the temperature-dependent electron heat capacity defined as a product of the Sommerfeld constant (γ) and the electron temperature ($T_{e}$), $C_{e}$ = γ$T_{e}$, $C_{l}$ the lattice heat capacity taken at 298 K, *G* the electron–phonon coupling factor, *k_e_* the thermal conductivity of electrons, *R* the reflectivity, $t_{p}$ the laser pulse duration defined as full width at half maximum of the laser pulse, $\delta_{s}$ the optical penetration depth, x the depth measured from the front surface, and β = 4ln(2).^34,36–38^ Upon the excitation, the lattice temperature is calculated to increase up to ∼360 K within 10 ps, which is in good agreement with the obtained timescale of 6.5 ps. During this thermalization process, the volume of individual lattices is preserved; thus, on a macroscopic scale, the surface area does not change, which is proven as an absence of morphological change on the same timescale. Second, the set of time-dependent Bragg spot positions reflects the change in lattice parameters in reciprocal space. The separation decrease in conjugate Bragg peaks in time in Fig. 3(d) reveals the expansion of the cubic lattice in ∼300 ps following its thermalization. The temperature after the thermalization followed by the expansion was estimated to be 393 ± 6 K from the time-dependent contraction of conjugate Bragg spots as much as −0.0013% on linear expansion with the thermal expansion coefficient, $\alpha_{L}$ = 14 × 10^−6^ K^−1^, which is similar to the lattice temperature after the thermalization from the TTM. In Fig. 3(f), we pictorially summarize our observation on the structural dynamics of the single crystalline gold membrane upon fs photoexcitation combining time-resolved image (macroscopic) and diffraction (microscopic) analyses. The results show the rapid temperature rise in several ps due to the heat transfer via e-ph coupling, in agreement with the initial heating time obtained from the simulation, and the morphology warping occurs in up to hundreds of ps via atomic displacements within the structure as it follows the collective lattice expansion.

In conclusion, with integrating a direct electron-detection camera of high sensitivity, low-repetition stroboscopic imaging at ≤10 kHz using the pulsed photoelectron beam driven by fs optical pulses has been made possible. The implication of this result is that UEM experiments can be set up to host a wider selection of specimen via controlling the interval between incident excitation pulses as long as 100 *μ*s, which is intended to allow for the full relaxation of the specimen before the irradiation of the successive pulse on it. Ongoing efforts are currently being made also to maximize the coherent beam current by optimizing the photoelectron-generation condition.

See supplementary material for the comparison of bright-field micrographs of single-crystalline gold membranes in three different modes of the K2 Summit direct detector using a pulsed electron beam at the repetition rates of 5 kHz and 10 kHz.
